# Unmasking metabolic disruptors: The NEMESIS project's quest for Novel Biomarkers, Evidence on Adverse Effects, and Efficient Methodologies

**DOI:** 10.12688/openreseurope.18439.2

**Published:** 2025-12-09

**Authors:** Henriikka Hakomäki, Sini Pitkänen, Anna-Liisa Levonen, Paavo Honkakoski, Dario Greco, Nicoletta D'Alessandro, Laura Aliisa Saarimäki, Susana Viegas, Cristina Godinho, Nanna Fyhrquist, Emma Wincent, Volker M Lauschke, Janne Hukkanen, Jukka Hakkola, Ludovic Vallier, Vittorio Fortino, Antreas Afantitis, Toshiaki Sawatani, Tereso J Guzman, Miriam Cnop, Tim Nawrot, Sophia Harlid, Marie-Therese Vinnars, Adonina Tardon, Joan O Grimalt, Jenni Küblbeck, Jaana Rysä

**Affiliations:** 1 A.I. Virtanen Institute for Molecular Sciences, University of Eastern Finland, Kuopio, Neulaniementie 2, 70211, Finland; 2 School of Pharmacy, University of Eastern Finland, Kuopio, Yliopistonrinne 3, 70211, Finland; 3 FHAIVE, Faculty of Medicine and Health Technology, Tampere University, Tampere, Arvo Ylpön katu 34, 33520, Finland; 4 NOVA National School of Public Health, Public Health Research Centre, Comprehensive Health Research Center, CHRC, REAL, CCAL, NOVA University Lisbon, Lisbon, Av. Padre Cruz, 1600-560, Portugal; 5 Institute of Environmental Medicine, Karolinska Institutet, Stockholm, Nobels väg 13, 171 77, Sweden; 6 Public Health Sciences, Department of Health, Karlstad University, Karlstad, Varmland County, Universitetsgatan 2, 651 88, Sweden; 7 Department of Physiology and Pharmacology, Karolinska Institutet, Stockholm, Solnavägen 1, 171 77, Sweden; 8 Center for Molecular Medicine, Karolinska Institutet and University Hospital, Stockholm, Visionsgatan 18, L8, 171 76, Sweden; 9 Dr Margarete Fischer Bosch Institute of Clinical Pharmacology, Stuttgart, Baden-Württemberg, Auerbachstraße 112, 70376, Germany; 10 University of Tübingen, Tübingen, Baden-Württemberg, Geschwister-Scholl-Platz, 72074, Germany; 11 Research Unit of Biomedicine and Internal Medicine, University of Oulu, Oulu, POB 5000, FI-90014, Finland; 12 Medical Research Center Oulu, University of Oulu and Oulu University Hospital, Oulu, POB 5000, FI-90014, Finland; 13 BIH Center for Regenerative Therapies, Berlin Institute of Health at Charite, Berlin, Föhrer Str. 15, 13353, Germany; 14 Max Planck Institute for Molecular Genetics, Berlin, Ihnestraße 63, 14195, Germany; 15 Institute of Biomedicine, School of Medicine, University of Eastern Finland, Kuopio, Yliopistonranta 8, 70211, Finland; 16 NovaMechanics Ltd, Nicosia, Digeni Akrita 51, 1070, Cyprus; 17 ULB Center for Diabetes Research, Universite Libre de Bruxelles, Brussels, CP618 Erasme, 1070, Belgium; 18 Division of Endocrinology, ULB Erasmus Hospital, Université Libre de Bruxelles, Brussels, Rte de Lennik 808, 1070, Belgium; 19 WEL Research Institute, Wavre, Avenue Pasteur 6, 1300, Belgium; 20 Centre for Environmental Sciences, Hasselt University, Hasselt, Agoralaan z/n, Gebouw D, 3590 Diepenbeek, Belgium; 21 Department of Public Health & Primary Care, Occupational & Environmental Medicine, KU Leuven, Leuven, Herestraat 49, 3001, Belgium; 22 Department of Diagnostics and Intervention, Oncology, Umeå University, Umeå, 901 87, Sweden; 23 Department of Clinical Sciences, Obstetrics and Gynecology, Umeå University, Umeå, 901 85, Sweden; 24 Health Research Institute of Asturias (FINBA-ISPA) and University of Oviedo, Oviedo, Asturias, Av. del Hospital Universitario, s/n, 33011, Spain; 25 Institute of Environmental Assessment and Water Research, IDAEA-CSIC, Barcelona, 18 26, Carrer de Jordi Girona, 18-26, 08034, Spain

**Keywords:** metabolism disrupting chemicals, endocrine disruption, chemical safety, new approach methodologies, metabolic health, risk assessment, liver steatosis, diabetes

## Abstract

Metabolism disrupting chemicals (MDCs) elicit negative effects on metabolically active organs such as the liver and the pancreas, altering normal metabolic processes. Chemicals that are known, or suspected MDCs include compounds found in everyday consumer products and food, making low-dose, continuous exposure inevitable for humans. Through the discovery of chemically induced metabolic disruption, a concern has surfaced whether and how MDCs impact human health and the development of metabolic diseases. This has accelerated research around the topic, and it has been found that exposure to MDCs is linked to increased incidence of metabolic diseases including obesity and liver steatosis. Effective regulatory action is hindered by the lack of accurate methods to identify MDCs. The NEMESIS project addresses this regulatory gap by investigating the mechanisms through which MDCs cause metabolic disruption. The project aims at identifying novel biomarkers of exposure and link exposure to disease outcomes. As chemical toxicity testing is rapidly moving towards new approach methodologies (NAMs), NEMESIS promotes non-animal methodologies by employing state-of-the-art *in vitro* methods, epidemiological data, systems biology approaches, and seeks to replace mammalian *in vivo* experiments with alternative models. By understanding mechanisms of MDC-induced metabolic health effects, and through the development of reliable effect biomarkers and testing strategies, the NEMESIS project aims to facilitate more effective regulatory measures to improve and protect the health and well-being of EU citizens. The project is particularly focused on maximizing its impact through effective dissemination and communication efforts, to ensure that the project’s message and results reach a broad audience and are tailored to different population groups. These actions will improve the risk assessment of MDCs and ensure that the EU citizens are informed and protected from the harmful effects of MDCs and can adapt their consumer patterns and behaviors to prevent exposure.

## Disclaimer

Funded by the European Union. Views and opinions expressed are however those of the authors only and do not necessarily reflect those of the European Union or the European Health and Digital Executive Agency (HaDEA). Neither the European Union nor the granting authority can be held responsible for them.

The views expressed in this article are those of the authors. Publication in Open Research Europe does not imply endorsement of the European Commission.

## Abbreviations

4βHC        4β-hydroxycholesterol

ATP          Adenosine triphosphate

CAR         Constitutive androstane receptor

EU            European Union

MDC        Metabolism disrupting chemicals

NAMs       New approach methodologies

NR            Nuclear receptor

MASLD    Metabolism dysfunction-associated steatotic liver disease

PFAS         Per- and polyfluoroalkyl substances

PXR          Pregnane X receptor

QSAR       Quantitative structure-activity relationship

ROS          Reactive oxygen species

## Introduction

Metabolic diseases represent a major public health concern in today’s world ( Chew *et al.*, 2023). This umbrella term includes diseases such as obesity, type 2 diabetes, metabolic dysfunction-associated steatotic liver disease (MASLD; previously known as non-alcoholic fatty liver disease), and dyslipidemia. These diseases arise from dysregulated metabolic functions in the human body. Beyond their impact on individual morbidity and mortality, metabolic diseases impose a vast global societal and financial burden in different sectors, underscoring the need for effective preventive measures.

In addition to the well-known risk factors like genetics, high-calorie diet, and sedentary lifestyle, environmental factors also negatively affect metabolic health ( Heindel *et al.*, 2017). Among these, chemically induced metabolic disruption is of particular concern. Known and suspected metabolism disrupting chemicals (MDCs) are practically everywhere, as they are found in a wide array of consumer products, including foods, packages, and cosmetics. Therefore, due to their extensive global use, exposure to MDCs is unavoidable, making research on their negative health effects crucial.

The mechanisms of chemically induced metabolic disruption are only partially understood. MDCs form a heterogeneous group of chemicals, and the body’s metabolic processes are complex and influenced by multiple factors. Different chemicals target different organs and metabolic processes, and exposure to MDCs varies greatly in terms of route of exposure, doses, mixtures, and duration of exposure, as well as their toxicokinetic behavior upon exposure. Therefore, more research is needed to better understand the underlying mechanisms and exposure scenarios to fully grasp the scope of MDC-induced toxicity.

Exposure to a variety of exogenous chemicals, in unknown quantities and mixtures, is an inevitable part of human life. It is the responsibility of chemical regulations to protect citizens from harmful chemicals and their adverse health effects. The European Union (EU) is committed to tackling the challenges that hinder the identification and evidence-based regulation of MDCs by investing in their research. Challenges that persist involve reliable identification of these chemicals, development of feasible non-animal test methodologies, understanding mixture effects, and identifying sensitive windows of exposure.

The NEMESIS consortium, funded by the EU’s Horizon Europe Programme aims to give solutions to these challenges and provide knowledge that supports evidence-based regulation of MDCs. Here, we present the framework of the NEMESIS project from its rationale to the project structure and execution.

## What is known about chemical-induced metabolic disruption

**Figure 1.**
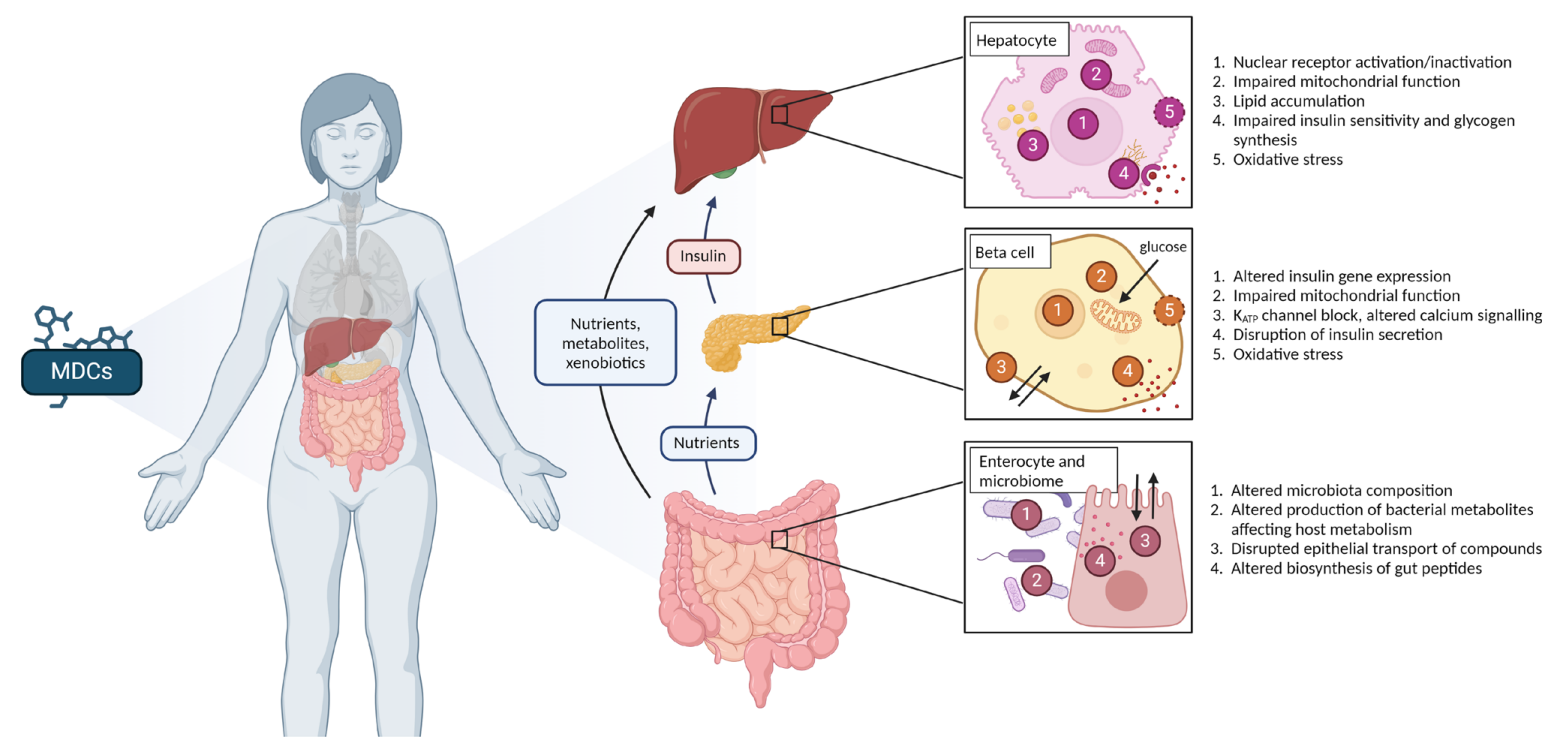
Proposed mechanisms by which metabolism disrupting chemicals (MDCs) disturb the metabolic processes at the level of the target organs studied in the NEMESIS-project: liver, pancreas, and gut. Created in Biorender.

MDCs can promote metabolic disruption through diverse mechanisms that are not fully understood ( Nadal *et al.*, 2017). Target organs for MDCs include metabolically active organs, such as the liver, placenta, brown and white adipose tissue, skeletal muscle, gut, and pancreas. MDCs can also affect distal organs responsible for the biosynthesis and secretion of key mediators such as hormones and cytokines ( Nadal *et al.*, 2017). In the NEMESIS project, the focus will be on liver, pancreas, and the gut (including the microbiota). The known mechanisms of action of MDCs on these target organs are presented in Figure 1. In addition, the MDC effects on early life will be studied. Prenatal life is the most sensitive stage of human development to environmental pollutants, with a predominant maternal source that transfers the pollutants into the placenta and the fetus ( Vizcaino *et al.*, 2014)

The **liver** is a central organ for the metabolism of xenobiotics and macronutrients. In the liver, disrupted carbohydrate and lipid homeostasis may develop into a progressive liver disease known as MASLD. Nuclear receptors (NR) are considered major mediators of hepatic metabolic effects of MDCs) ( Balaguer *et al.*, 2019; Fritsche *et al.*, 2023). The liver expresses 47 out of the 48 NRs encoded in the human genome and structural data indicate that MDCs bind to a considerable number of these ( Tan *et al.*, 2021). MDCs can also affect metabolism by impacting central cellular organelles such as mitochondria either directly or indirectly via transcriptional regulation ( Marroqui *et al.*, 2018; Reddam *et al.*, 2022). For example, MDCs have been shown to alter mitochondrial membrane potential, impair production of adenosine triphosphate (ATP), and increase the production of reactive oxygen species (ROS) ( Marroqui *et al.*, 2018; Reddam *et al.*, 2022). Thus, understanding the influence of MDCs on the adult liver and on liver development is essential. However, studies on primary liver cells, especially hepatocytes which represent the main functional unit of the liver, remain challenging. To address this limitation, taking advantage of the hepatocyte spheroids generated from primary tissue and culturing them in 3D culture conditions is the state-of-the-art ( Bell *et al.*, 2016). The resulting cells remain functional and provide an advantageous platform for analyzing the impact of MDCs on hepatocytes’ metabolic functions.

The **endocrine pancreas** plays a key role in the regulation of metabolism through hormone secretion. Insulin secretion by beta cells in the islets of Langerhans is essential to maintain blood glucose levels in a normal range and modulate lipid and protein biosynthesis due to its anabolic effects. MDCs can negatively affect functional beta cell mass and thereby perturb metabolic homeostasis. Dysfunction of beta cells may be due to alterations of organelle function, ion channel activity, cellular oxidative state, and other mechanisms. This will lead to impaired insulin secretion, hyperglycemia, and the development of type 2 diabetes ( Eizirik *et al.*, 2020).

Pancreatic beta cells are rather difficult to access. Organ donor human islets, the gold standard for diabetes research, only occasionally become available for research, and islets from type 2 diabetic donors are seldomly isolated ( Eizirik & Cnop, 2012). Over the past decade, human beta cell lines, such as EndoC-βH1 and -βH5, have been developed, representing useful experimental models ( Blanchi *et al.*, 2023; Ravassard *et al.*, 2011). In 2007, induced pluripotent stem cell (iPSC) technology was applied to human cells ( Takahashi *et al.*, 2007). This method of producing iPSCs from somatic cells has paved the way for studying diseases and performing chemical screens *in vitro* in a species-specific manner. Leveraging human iPSCs technology and differentiating these cells into primary islet-like aggregates, provides a unique opportunity for human islet research with more convenient access to this human biological material ( Cosentino *et al.*, 2018; De Franco *et al.*, 2020; Demine *et al.*, 2020; Fantuzzi *et al.*, 2022; Gorgogietas *et al.*, 2023; Igoillo-Esteve *et al.*, 2020; Lytrivi *et al.*, 2021; Oshima *et al.*, 2020). These different human beta cell models are close enough to organ donor human islets to study the impact of MDCs on beta cell function.

The role of the **gastrointestinal microbiota** in metabolic diseases and the chemical-induced metabolic disruption is an understudied, but emerging area of research. It is postulated that MDCs may modify the composition and function of the microbiota and bring about adverse health outcomes. The gut microbiota is increasingly recognized as a key contributor to the development of metabolic disorders, due to its ability to interact with the host by producing a wide range of metabolites from both external substrates and the host's own compounds. Specific classes of microbiota-derived metabolites, such as bile acids, short-chain fatty acids and tryptophan metabolites, have been implicated in the pathogenesis of metabolic disorders ( Agus *et al.*, 2021).

Moreover, the complex interaction between the gut microbiota and the host immune system makes it a highly intriguing area of exploration. Dysbiosis has been associated with low-grade inflammation, a common feature of obesity and type 2 diabetes. This inflammation may be driven by immune responses that are influenced by microbial signals. Additionally, increased permeability of the intestinal epithelium, caused by various factors such as diet, medications, infections, stress, or environmental toxins including MDCs, allows bacterial endotoxins to enter the bloodstream, triggering further inflammation and metabolic disturbances ( de Vos *et al.*, 2022). 

The gastrointestinal tract acts as a primary entry point for MDCs. Some of these chemicals are metabolized by microbial flora, which can either enhance or reduce their toxicity. A portion of the modified MDCs is transported to the liver, where they undergo conjugation during phase II metabolism, and are then secreted into the bile. When these compounds re-enter the small intestine, they are further deconjugated, which can restore the original chemicals or generate new, toxic metabolites ( Street *et al.*, 2023).

The microflora can metabolize MDCs into both biologically active and inactive forms. In turn, MDCs can either promote or inhibit the growth of certain bacteria, resulting in dysbiosis and disruptions in the host system. Experiments with mice exposed to bisphenol A have shown dysbiosis and inflammation occurring before the onset of obesity-related traits ( Lai *et al.*, 2016; Malaisé *et al.*, 2017). Although animal studies have often linked perinatal exposure to MDCs with metabolic disorders, the obesogenic effects of MDCs in humans remain debatable. The mechanisms by which MDC exposure may contribute to metabolic disorders, especially through interactions with the gut microbiome, are not yet fully understood. Unraveling these host-microbiota interactions is a significant public health challenge.

Functioning **placental** metabolism is crucial for fetal development and is closely linked to maternal metabolism. On top of the growing population of pregnant women being obese or having diabetes prior to pregnancy, metabolic disorders such as gestational diabetes and preeclampsia may arise during pregnancy ( Bedell *et al.*, 2021; Jiang *et al.*, 2022; Louwen *et al.*, 2024). These conditions are associated with improper placental development, which can lead to severe outcomes including placental infarctions, fetal growth restriction, and intrauterine fetal death. Numerous animal studies have examined the effects of MDCs — including bisphenols, polychlorinated biphenyls, and perfluorinated compounds — on placental development. However, these studies often lack relevant exposure conditions, making it challenging to apply their findings to humans ( Gingrich *et al.*, 2020). To remedy this, improved access to human biomonitoring data is essential. In addition, more sophisticated exposure assessments and evaluations of chemical mixtures will be important to unravel the links between MDC exposures, placental metabolic function, complex pregnancy disorders, and early life development.

NEMESIS aims to uncover the underlying mechanisms of MDC-induced metabolic disruption by integrating *in vitro* methodologies, mouse and zebrafish models, along with human cohorts, using a systems biology framework for more comprehensive understanding. By combining findings from multiple sources, the project seeks to unravel the complex interactions between MDC exposure and metabolic effects. However, the key challenges in MDC research include low-dose and mixture effects, non-linear dose response relationships, and an unknown lag time from exposure to disease. To gain better understanding, it is crucial to investigate diverse exposure scenarios, gather data from multiple levels of a biological system (cell, organ, whole organism, transcriptome, metabolome), and identify and assess the sensitive windows of exposure, recognizing the possibility of transgenerational effects

## The way forward

The NEMESIS project builds upon current regulatory practices ( EU Regulation No 1907/2006 and 2017/2100, Andersson *et al*., 2018) as well as previous research, method development and regulatory application efforts of e.g., the H2020 projects EDCMET, Goliath, and Oberon, focused on understanding MDCs. Traditional regulatory framework assesses the safety of chemicals individually, substance-by-substance, yet real-world exposure always involves mixtures of these chemicals. This is also how research has been conducted, as it is important to unravel the effects of single substances before assessing their roles in complex mixtures. However, there is limited understanding on the causality of mixture exposures and associated adverse health effects despite their clinical relevance.

Moving forward, the NEMESIS project aims to advance the understanding of MDCs not only by assessing how individual substances cause adverse health effects, but also by investigating the causal relationships between mixture exposures and metabolic disruption outcomes, through modelling of multilevel experimental data. This will provide insights on how the risk assessment process could and should be refined. Through this approach, the project seeks to gain insight into MDCs that will aid in the development of more comprehensive risk assessment and regulatory strategies to address the challenges of MDC-mixtures. This evidence is exactly what regulators and public-health agencies need to update hazard identification, risk assessment and risk-management decisions across EU chemical safety and related consumer/food policies.

## Ambition and methodology of the NEMESIS project

The ambition of the NEMESIS project is to enhance understanding of MDCs, among the scientific community and regulators, but also among EU citizens. By engaging and educating citizens, through a co-creation approach and the development of clear and accessible communication materials, the project seeks to encourage informed health-promoting behaviors in everyday life, ultimately reducing upstream determinants of metabolic diseases through citizen actions.

To address the persisting scientific uncertainties regarding MDCs for regulatory purposes, and to inform and engage citizens effectively, the NEMESIS project is structured around key objectives that guide the project towards its aims:

i) Generate a unified approach to identify MDCs causing adverse metabolic effects, through identifying MDC-mediated metabolic effects, susceptible groups, and by adopting alternative test methods to animal testing.
ii) Increase mechanistic understanding of adverse metabolic effects of MDCs in liver, pancreas, and microbiota, by providing mechanistic data, and assessing their dose-response relationships, and causality between exposure and adverse effects.
iii) Provide data to enable MDCs exposure characterization by assessing levels of MDCs in Southern, Central, and Northern Europe from biomonitoring samples and identify novel predictive biomarkers of metabolic disruption.
iv) Integrate, curate, standardize, and harmonize existing as well as newly generated *in silico* and toxicogenomic data regarding MDCs. Provide computational tools to enable the exploitation of existing and *de novo* data generated within the project, helping to model the causal relationships between MDC exposure and adverse health outcomes.
v) Refine and improve risk assessment of MDCs, through the integration of the adverse outcome pathways (AOP) framework with toxicogenomics data for metabolic disruption, promoting the use of integrated approaches to testing and assessment, and endorsing a more holistic risk assessment of chemicals from substance-by-substance approach to regulation of groups of related chemicals and mixtures of MDCs.
vi) Increase knowledge and awareness on metabolic adverse effects of MDCs and promote preventive health behaviors, through wide dissemination of evidence-based, co-created communication materials through a dedicated website and social media accounts targeting the general population and relevant stakeholders (e.g., patients’ and consumers’ associations, healthcare professionals, public health, environmental and food safety agencies, etc.). Citizens will also be engaged in the development of chemical policies by assessing their acceptability of different policy measures.

Within the NEMESIS project, our focus is on three chemical groups of significant concern: bisphenols, phthalates, and per- and polyfluoroalkyl substances (PFAS). These chemicals are known to disrupt metabolic homeostasis and are widely present in consumer products and food, leading to continuous low-dose exposure for EU citizens.

Human biomonitoring data is collected from several European population-based cohorts. This data will help us to establish the levels of MDCs across different regions, identify the most common MDC mixtures, assess the effectiveness of existing chemical policies, and identify novel biomarkers of metabolic disruption. Importantly, the human biomonitoring data will define the landscape of real-world mixture exposure, guiding our selection of the relevant mixtures to be studied in the project. Novel and easily applicable biomarkers of MDC exposure and action are required to fulfill the ambition of NEMESIS. As mentioned, many MDCs target NRs expressed in the liver, and we will screen the selected MDC classes, their metabolites and microbiota-derived compounds with a prevalidated battery of NR assays. As an example, especially pregnane X receptor (PXR) and constitutive androstane receptor (CAR) are being activated by a multitude of MDCs ( Kojima *et al.*, 2011; Küblbeck *et al.*, 2020). Circulating 4β-hydroxycholesterol (4βHC), formed in liver under the control of PXR and CAR, can be highly elevated by exposure to PXR/CAR agonists ( Hukkanen & Hakkola, 2020). We will leverage the prior knowledge to establish 4βHC-based indices as biomarkers of 1) exposure to MDC mixtures and 2) MDC-mediated metabolic disruption.

Increasing mechanistic understanding of chemically induced metabolic disruption is the core of the project. We focus on studying target organs, such as liver, intestine and endocrine pancreas, using an array of different methods. These include advanced *in vitro* methods, such as 3D primary human cell culture models that recapitulate human tissue functions for extended periods of time, thereby offering possibilities to conduct chronic exposure studies in physiologically relevant contexts ( Youhanna *et al.*, 2022). In addition, we will study the effect of MDCs on fetal development by using human iPSCs derived liver bud which mimic early organogenesis of the liver *in vitro* ( Wesley *et al.*, 2022). This approach will reveal how MDCs not only impact fetal organ development but also trigger the early onset of adult diseases. Further, optimized cell-based assays are also employed to investigate species-specific NR activation and mitochondrial dysfunction caused by MDCs.

Recognizing that metabolic homeostasis involves complex interactions among multiple organs, we use non-mammalian (zebrafish) and mammalian (mouse) vertebrate models to uncover how MDCs cause metabolic disruption in an intact organism. Mechanistic understanding of the *in vivo* effects such as hormonal signalling and other intertissue communications are essential for proper prediction of metabolic disruption. The AOP framework, a regulatory accepted model, supports this mechanistic understanding by providing a structured way to organize biological processes into linked sequence of key events ( Ankley *et al.,* 2010) across multiple biological scales, from the initial molecular interaction to the resulting phenotypic adverse metabolic outcomes. While AOPs are inherently theoretical, the recent development of gene set annotations of human-relevant AOPs allows us to map omics responses from different models onto established mechanistic AOPs, thereby enhancing the interpretability of NAMs and supporting cross-species extrapolation ( Saarimäki *et al.,* 2023). Thus, within NEMESIS project, we leverage the AOP framework together with toxicogenomic data to interpret the mechanism underlying MDCs adverse effects. We aggregate MDCs omics related datasets and interpreted them within the AOP framework to extract the mechanisms of action that link exposure with the adverse metabolic effects. Additionally, the integration of multiple data sources contextualizes the extracted mechanisms and supports the prioritization of the most relevant mechanisms based on exposure scenarios. In parallel, the IATA framework guides the structured integration of these diverse evidence streams, enabling a comprehensive, weight-of-evidence evaluation of MDC-induced metabolic disruption. Furthermore, the *in vivo* models allow detection of complex consequences of metabolic disruption such as cardiovascular outcomes in response to disturbance of liver lipid metabolism. By comparing effects of exposure during different life stages and dietary regimens, a deeper understanding of sensitive windows of exposure and synergies with lifestyle factors is achieved. This approach allows us to validate predictive non-animal and non-mammalian models, facilitating the transition towards relying on NAMs.

In addition to controlled experiments, NEMESIS utilizes real-life birth cohorts to address real-world scenarios. Since the placenta plays a pivotal role in fetal development, any structural or molecular changes including metabolic alterations in the placenta can have profound implications for both immediate and long-term health. Therefore, markers related to inflammation, endocrine function, and metabolic health, alongside indicators of placental function, are highly relevant. Furthermore, assessing early-life developmental outcomes, such as ultrasound measurements of in utero growth, provides valuable insights into future health outcomes, including metabolic disorders and obesity in child- and adulthood. This holistic approach aims to enhance our understanding of how early-life conditions influence lifelong health trajectories.

Throughout the project, various omics data types are collected to reveal the changes inflicted by the exposure to MDCs. An exciting aspect of the NEMESIS project is the development of computational tools to integrate and analyze omics data alongside e.g., *in silico*-generated quantitative structure-activity relationship (QSAR) data, with a special focus on dose-response relationships. The NEMESIS project will provide a systems biology framework for modeling MDC-mediated metabolic effects, starting from the analysis of NEMESIS-generated multi-omics data of MDC exposures. This data will be represented as networks of molecular features and pathways, characterizing the link between molecular initiating events, genes dysregulated by MDC exposure, genes associated with metabolic diseases, and available targeted toxicological effects. Mechanistic information generated in this way will be exploited to inform on biomarkers of MDC-mediated effects and to develop a machine learning-based framework for predicting the targeted toxicological or adverse health effects.

In the scope of the NEMESIS project, a user-friendly cloud platform will be developed to provide access to predictive models for hazard prediction and risk assessment of MDCs. This platform, built on advanced cheminformatics technologies, will streamline complex scientific calculations and enhance accessibility for researchers. Drawing expertise gained from developing the NanoSolveIT platform, the NEMESIS microservices will incorporate software such as Isalos Analytics, Enalos Tools, R, KNIME, and Python ( Papadopoulou *et al.*, 2021; Stylianaki *et al.*, 2024).

The NEMESIS project is committed to maximizing its impact and the uptake of its results in the EU and beyond. In addition to the above-described open access platform, website and scientific conferences, NEMESIS will organize public workshops and also collaborate with the ENKORE Cluster to communicate its results and achievements to project stakeholders. Furthermore, the project consortium includes experts in social and behavioral sciences and will use participatory and co-creation methods for the development and testing of tailored communication strategies and materials on MDC exposure and risk, regulatory strategies, and health protecting behaviors. Through the engagement of the general public, including populations at increased risk associated with exposure to MDCs, the project will allow an in-depth understanding of the drivers and barriers of preventive health behaviors’ adoption, that is key for evidence-based health promotion efforts. Different communication formats (infographics, short videos, etc.) will be developed to enhance citizens’ understanding of harmful environmental chemicals and promote adherence to health-promoting behaviors. These will be widely disseminated through a dedicated website and social media. This holistic approach ensures that the benefits of our research efforts extend to the society as a whole and help to protect public health.

## Conclusions

MDCs represent a prevalent challenge to human health as their wide use e.g., in consumer products results in unavoidable, continuous exposure to these chemicals. Because exposure to MDCs has been linked to increased incidence of metabolic diseases, the NEMESIS project aims to address critical gaps in understanding how this happens, to pave the way for more effective and evidence-based regulatory measures. Within the project, we will provide insights from biomonitoring data on MDC levels and mixtures across different regions, enhance the understanding of how MDCs impact human health, increase mechanistic understanding of chemically induced metabolic disruption, develop predictive computational tools to predict outcomes of exposure, and communicate the project results effectively to the public to raise awareness and promote health-promoting behaviors. By integrating the project’s findings into regulatory measures, the NEMESIS project aims to protect and improve the public health of the EU citizens and beyond.

## Ethics and consent

Ethical approval and consent were not required.

## Data Availability

No data are associated with this article.
