# Supplementary material for: Unmasking metabolic disruptors: The NEMESIS project's quest for Novel Biomarkers, Evidence on Adverse Effects, and Efficient Methodologies
Source: Open Res Eur. 2025 Dec 9;4:194. Originally published 2024 Sep 5. [Version 2] doi: 10.12688/openreseurope.18439.2 (PMC12749563; doi:10.12688/openreseurope.18439.2)
Supplement: Peer review history

# Reviewer report: Reviewer response for version 2

**DOI:** 10.21956/openreseurope.23764.r65790

**Published:** 2025-12-29

**Authors:** Sofie Christiansen

**Affiliations:** 1 National Food Institute, Technical University of Denmark, Kongens Lyngby, Denmark

My comments have been fully addressed by the authors.

Where applicable, are recommendations and next steps explained clearly for others to follow? (Please consider whether others in the research community would be able to implement guidelines or recommendations and/or constructively engage in the debate)

Partly

Does the article adequately reference differing views and opinions?

Partly

Are all factual statements correct, and are statements and arguments made adequately supported by citations?

Partly

Is the rationale for the Open Letter provided in sufficient detail? (Please consider whether existing challenges in the field are outlined clearly and whether the purpose of the letter is explained)

Yes

Is the Open Letter written in accessible language? (Please consider whether all subject-specific terms, concepts and abbreviations are explained)

Yes

Reviewer Expertise:

Endocrine disruption and regulatory toxicology

I confirm that I have read this submission and believe that I have an appropriate level of expertise to confirm that it is of an acceptable scientific standard.

# Reviewer report: Reviewer response for version 1

**DOI:** 10.21956/openreseurope.19927.r61864

**Published:** 2025-10-22

**Authors:** Sofie Christiansen

**Affiliations:** 1 National Food Institute, Technical University of Denmark, Kongens Lyngby, Denmark

General Assessment

 This open letter outlines the rationale, goals, and methodology of the NEMESIS project, which tackles the regulatory and scientific challenges posed by metabolism-disrupting chemicals (MDCs). The initiative is timely and aligns with the EU’s growing focus on endocrine and metabolic disruptors. NEMESIS aims to develop biomarkers, apply New Approach Methodologies (NAMs), and raise public awareness.

 Strengths

- NEMESIS supports EU priorities, especially the transition to NAMs and non-animal testing. It addresses critical gaps in MDC identification, including mixture toxicity, low-dose effects, and vulnerable exposure windows.
- The use of human biomonitoring and real-life cohorts strengthens the translational relevance for regulatory risk assessment.
- NEMESIS employs cutting-edge methods, including multi-omics, systems biology, and advanced in vitro models (e.g., 3D hepatocyte spheroids, iPSC-derived islets).
- The mechanistic focus across organs (liver, pancreas, gut, placenta) is comprehensive and well-justified.
- The letter cites MDC literature and relevant studies on CAR/PXR pathways.

Weaknesses

- The letter lacks references to specific EU regulatory frameworks (e.g., REACH) and how NEMESIS outputs could improve regulation risk assessment.
- The limitation of Biomonitoring data is not mentioned. Biomonitoring data can indicate exposure to chemicals but cannot establish causality, or direct links to health outcomes.
- While dissemination is mentioned, mechanisms for regulatory endorsement or stakeholder engagement are not clearly defined.
- Figure 1 is conceptually useful but lacks a clear legend linking it to NEMESIS.
- The former H2020 project GOLIATH, coordinated by Juliette Legler, developed MDC-specific Integrated Approaches to Testing and Assessment (IATA), validated nuclear receptor assays, and engaged with the OECD test guideline program.
  - The letter omits direct references to GOLIATH publications, which is a notable oversight given the thematic and regulatory overlap.
  - One publication Legler et al. (2020) ( https://beatinggoliath.eu/) could be referred to in the NEMESIS letter, e.g., under the heading “the way forward”.

Recommendations

- Detail how adverse outcome pathways (AOPs) and IATA frameworks are integrated into the project it is only briefly mentioned as aim number 5.
- Clarify how NEMESIS findings will influence chemical safety legislation, consumer protection, and public health.

Where applicable, are recommendations and next steps explained clearly for others to follow? (Please consider whether others in the research community would be able to implement guidelines or recommendations and/or constructively engage in the debate)

Partly

Does the article adequately reference differing views and opinions?

Partly

Are all factual statements correct, and are statements and arguments made adequately supported by citations?

Partly

Is the rationale for the Open Letter provided in sufficient detail? (Please consider whether existing challenges in the field are outlined clearly and whether the purpose of the letter is explained)

Yes

Is the Open Letter written in accessible language? (Please consider whether all subject-specific terms, concepts and abbreviations are explained)

Yes

Reviewer Expertise:

Endocrine disruption and regulatory toxicology

I confirm that I have read this submission and believe that I have an appropriate level of expertise to confirm that it is of an acceptable scientific standard, however I have significant reservations, as outlined above.

## References

[1] : The GOLIATH Project: Towards an Internationally Harmonised Approach for Testing Metabolism Disrupting Compounds. *International Journal of Molecular Sciences* .2020;21(10) : 10.3390/ijms21103480 10.3390/ijms21103480 PMC727902332423144

# Response

**Published:** 2025-11-17

**Authors:** Henriikka Hakomäki

**Affiliations:** A.I. Virtanen Institute for Molecular Sciences, University of Eastern Finland - Kuopio Campus, Kuopio, North Savo, Finland

Dear **Sofie Christiansen**, We wish to warmly thank you for taking the time to review our manuscript, *“ **Unmasking metabolic disruptors: The NEMESIS project's quest for Novel Biomarkers, Evidence on Adverse Effects, and Efficient Methodologies**”* submitted to Open Research Europe. We greatly value your comments and constructive suggestions, which have helped us to improve the clarity of our manuscript. We hope that our revision and responses clarify the points that were raised.

**Reviewer comment:** The letter lacks references to specific EU regulatory frameworks (e.g., REACH) and how NEMESIS outputs could improve regulation risk assessment.

***Authors’ response:** We thank the reviewer for their comment. We have modified the text accordingly to clarify how NEMESIS outputs will improve risk assessment, and added relevant references mentioned.*

**Reviewer comment: **The limitation of Biomonitoring data is not mentioned. Biomonitoring data can indicate exposure to chemicals but cannot establish causality, or direct links to health outcomes.

***Authors’ response:** We completely agree with the Reviewer. While biomonitoring provides valuable information about the presence and levels of chemicals in human tissues or fluids, it does not establish causality. It indicates exposure but cannot determine whether a specific chemical caused a particular health effect. In NEMESIS, biomonitoring is collected from several European population-based cohorts and used to establish the levels of MDCs across different regions. This will help us to identify the most common MDC mixtures for experimental studies. The novel measurements of exposure levels can give us valuable information on the effectiveness of existing chemical policies. In addition, biomonitoring supports the discovery of new biomarkers by integrating omics data and exposure profiles to identify molecular signatures associated with MDC-related metabolic effects. When biomonitoring data is combined with epidemiological, toxicological, and mechanistic studies it can be used to assess health risks of the chemicals.*

**Reviewer comment: **While dissemination is mentioned, mechanisms for regulatory endorsement or stakeholder engagement are not clearly defined.

***Authors’ response:** This has been shortly expanded in the paragraph describing how NEMESIS will maximise the uptake of its results in the EU and beyond.*

**Reviewer comment: **Figure 1 is conceptually useful but lacks a clear legend linking it to NEMESIS.

***Authors’ response: **Thank you for the notion, we have modified the legend accordingly.*

**Reviewer comment: **The former H2020 project GOLIATH, coordinated by Juliette Legler, developed MDC-specific Integrated Approaches to Testing and Assessment (IATA), validated nuclear receptor assays, and engaged with the OECD test guideline program. The letter omits direct references to GOLIATH publications, which is a notable oversight given the thematic and regulatory overlap.

***Authors’ response: **We thank the reviewer for pointing out the lack of acknowledging the prior projects working on the same theme. We have now included mentions to the three metabolic projects in the EURION Cluster (EDCMET, Goliath, and Oberon) and provided links to the respective websites where further information and publications can be found.*

**Reviewer comment: **Detail how adverse outcome pathways (AOPs) and IATA frameworks are integrated into the project it is only briefly mentioned as aim number 5.

***Authors’ response: **We thank the reviewer for this comment. We have now elaborated on the matter that was previously missing.*

**Reviewer comment: **Clarify how NEMESIS findings will influence chemical safety legislation, consumer protection, and public health.

***Authors’ response: **Indeed. We have now modified the manuscript according to these great suggestions.*  

 On behalf of the authors,

 Henriikka Hakomäki

# Reviewer report: Reviewer response for version 1

**DOI:** 10.21956/openreseurope.19927.r44235

**Published:** 2024-09-30

**Authors:** Ishfaq Ahmad Sheikh

**Affiliations:** 1 King Abdulaziz University, Jeddah, Makkah Province, Saudi Arabia

The article submitted by Hakomaki et al., highlights the main objectives of NEMESIS project on investigating the underlying molecular mechanism, developing predictive models to predict the health outcomes, and developing methodologies to understand the impact of Metabolic Disrupting Chemicals (MDCs) exposure on human health. The effective dissemination of results of this project is expected to mitigate their effects on human health. This will also improve the risk assessment of MDCs and ensure that the EU citizens are informed and protected from the harmful effects of MDCs.

**Conclusion**

 Overall, the open letter by Hakomaki et al., is presented well. Its clarity, relevance, and call to action make it a valuable contribution to the ongoing conversation in on MDC exposure. I recommend the letter for indexing.

Where applicable, are recommendations and next steps explained clearly for others to follow? (Please consider whether others in the research community would be able to implement guidelines or recommendations and/or constructively engage in the debate)

Partly

Does the article adequately reference differing views and opinions?

Yes

Are all factual statements correct, and are statements and arguments made adequately supported by citations?

Yes

Is the rationale for the Open Letter provided in sufficient detail? (Please consider whether existing challenges in the field are outlined clearly and whether the purpose of the letter is explained)

Yes

Is the Open Letter written in accessible language? (Please consider whether all subject-specific terms, concepts and abbreviations are explained)

Yes

Reviewer Expertise:

Endocrine Disruption

I confirm that I have read this submission and believe that I have an appropriate level of expertise to confirm that it is of an acceptable scientific standard.

# Response

**Published:** 2025-10-22

**Authors:** Henriikka Hakomäki

**Affiliations:** A.I. Virtanen Institute for Molecular Sciences, University of Eastern Finland - Kuopio Campus, Kuopio, North Savo, Finland

Dear Ishfaq Ahmad Sheikh, We would like to sincerely thank you for taking the time to review our manuscript, *“Unmasking metabolic disruptors: The NEMESIS project's quest for Novel Biomarkers, Evidence on Adverse Effects, and Efficient Methodologies”* submitted to Open Research Europe. We truly appreciate your thoughtful evaluation and are grateful that you found the paper satisfactory. Your positive feedback is very encouraging and reinforces our confidence in the work’s clarity and contribution. Thank you again for your time, effort, and valuable support throughout the review process. On behalf of the authors,

 Henriikka Hakomäki
